# On the Organs of Vision

**Published:** 1860-09

**Authors:** 


ART. IX.—*On the Organs of Vision: their Anatomy and Physiology.* By THOMAS NUNNELEY, F.R.C.S.E., Lecturer on Surgery in the Leeds School of Medicine, Senior Surgeon to the Leeds General Eye and Ear Infirmary, etc. etc. London: John Churchill, 1858. 8vo., pp. 373.

THIS excellent monograph emanates from the pen of a careful and laborious investigator, and though written, as we learn from the preface, during the constant interruption and distraction of active practice, it may justly be regarded as a valuable addition to the literature of the anatomy and physiology of the eye. It is professedly an attempt to supply both the student and the busy practitioner of medicine with a reliable manual of reference concerning the descriptive and minute anatomy of the eye, the more interesting and important phenomena of vision, the ideas obtained by sight and its relation to intellectual man, and the nature and elementary laws of light in their connection with vision. In making this attempt, Dr. Nunneley has not contented himself with merely a judicious selection of material from all the acknowledged authorities of the day; on the contrary, he has evidently been at much pains to verify the facts of others, as far as possible, by actual observation. In his elaborate account of the anatomy of the eye, for example, he appears to have described nothing that he had not previously dissected. He informs us that night by night continuously for more than two years he has steadily worked at the difficult microscopic structure of the many ocular tissues in various creatures, using some hundreds of eyes, and making some thousands of drawings of the parts. The product of labors like these, the work under consideration, comes to us with an authority which no mere compilation could possibly claim.

The pathology and embryological history of the eye not coming within the author's design, have been purposely omitted.

The volume is divided into seven chapters, and is handsomely illustrated with a series of eight plates, some of which are colored, and one hundred and seventy-nine wood engravings.

The first chapter treats of sensation and the senses generally. In the second are discussed the feelings and ideas ascribed to, and derived from, the sense of vision. The third chapter is devoted to the consideration of light, and such of its laws as are immediately applicable to vision. Chapters IV., V., and VI. are wholly occupied with anatomical details of considerable importance not only in a practical, but also in a scientific aspect. Chapter IV., "On the Structure of the Human Eye and its Appendages," is in every respect worthy of commendation. It is by far the longest article in the book, occupying, as it does, nearly one-half the entire volume. In this chapter the author has brought together, in a systematic and lucid manner, many well-known statements relative to the anatomy of the eye, and associated with them the results of his own skillful investigations. These independent researches have led him, in some instances, to controvert the statements put forth by other observers. Thus the globe of the eye is described by most anatomists as being a spheroid, the diameter of which from before backward is somewhat greater than in any other direction. By some, the antero-posterior diameter is given at 11 lines, the transverse at only 10. Petit described the proportion of the antero-posterior to the transverse diameter as 136 to 135; Soemmering, as 10 to 9·5. Dr. Nunneley, on the contrary, maintains that the transverse diameter is broader than the antero-posterior.

"I am much inclined," he says, "to think whatever departure there is from a spherical figure is in the contrary direction to that which it has been so confidently and commonly stated to be, and that the opinion of the antero-posterior diameter being larger than the transverse has rather been formed from looking at a section where the projection of the smaller curve of the cornea, set in, as it is, to the somewhat flattened termination of the sclerotic, gives rise to the appearance of greater projection than to actual measurement of the two diameters. It would be difficult to give any optical reason why the antero-posterior diameter should be the largest, and analogy is decidedly against the supposition, for in all animals where there is any decided difference in the two diameters, the transverse is the greatest; in many instances the distance being very considerable."

This is the case in the eye of the ox, sheep, horse, pig, hare, squirrel, mysticete whale, turkey, Guinea fowl, grouse, partridge, common fowl, rook, swan, duck, goose, alligator, green turtle, toad, frog, basking shark, salmon, trout, greyling, dace, golden carp, eel, cod, haddock, whiting, mackerel, herring, halibut, etc. etc.

As our author's opinion on this subject is opposed to the statement so generally made, he has taken considerable pains in accurately measuring a large number of eyes of both men and women and of various animals; the latter almost immediately after death, most of them instantly the animal was killed. The tables of measurement which he gives in support of his views embrace as many as two hundred eyes. These tables are also of importance as showing the form and size of the cornea, and particularly in pointing out the situation in which the optic nerve enters the sclerotic, and its distance from the axis of the eye. It has long been known that this entrance is eccentric as regards the transverse axis; our author calls attention to the fact that it is also eccentric vertically, in almost as great a degree in man, and in many animals to a much greater extent,—the distance from the entrance of the optic nerve over the upper surface of the sclerotic to the top margin of the cornea being in man decidedly greater than it is along the under surface to the lower margin, while in some animals the contrary prevails.

This chapter also contains careful measurements of the lens, and other parts of the eye.

In chapters V. and VI. the anatomy of the eyes of recent and fossil animals is briefly described.

The physiology of vision constitutes the subject-matter of chapter VII. In this part of the work the reader will find, among other important topics, some highly interesting remarks upon parallactical aberration and the accommodation of the eye to distances.

In concluding this brief notice, we feel it incumbent upon us to commend the work of Dr. Nunneley to our professional brethren as a highly useful addition to their libraries.

